# The Impact of Telemedicine on Physicians’ After-hours Electronic Health Record “Work Outside Work” During the COVID-19 Pandemic: Retrospective Cohort Study

**DOI:** 10.2196/34826

**Published:** 2022-07-28

**Authors:** Katharine Lawrence, Oded Nov, Devin Mann, Soumik Mandal, Eduardo Iturrate, Batia Wiesenfeld

**Affiliations:** 1 Department of Population Health New York University Grossman School of Medicine New York, NY United States; 2 Department of Medicine New York University Grossman School of Medicine New York, NY United States; 3 Medical Center Information Technology New York University Langone Health New York, NY United States; 4 Department of Technology Management and Innovation New York University Tandon School of Engineering New York, NY United States; 5 Management Department New York University Stern School of Business New York, NY United States

**Keywords:** telemedicine, telehealth, eHealth, COVID-19, EHR, electronic health record, clinician workload, impact, transition, workload, cohort, retrospective, physician, efficient, doctor, health care professional, pandemic

## Abstract

**Background:**

Telemedicine as a mode of health care work has grown dramatically during the COVID-19 pandemic; the impact of this transition on clinicians’ after-hours electronic health record (EHR)–based clinical and administrative work is unclear.

**Objective:**

This study assesses the impact of the transition to telemedicine during the COVID-19 pandemic on physicians’ EHR-based after-hours workload (ie, “work outside work”) at a large academic medical center in New York City.

**Methods:**

We conducted an EHR-based retrospective cohort study of ambulatory care physicians providing telemedicine services before the pandemic, during the acute pandemic, and after the acute pandemic, relating EHR-based after-hours work to telemedicine intensity (ie, percentage of care provided via telemedicine) and clinical load (ie, patient load per provider).

**Results:**

A total of 2129 physicians were included in this study. During the acute pandemic, the volume of care provided via telemedicine significantly increased for all physicians, whereas patient volume decreased. When normalized by clinical load (ie, average appointments per day by average clinical days per week), telemedicine intensity was positively associated with work outside work across time periods. This association was strongest after the acute pandemic.

**Conclusions:**

Taking physicians’ clinical load into account, physicians who devoted a higher proportion of their clinical time to telemedicine throughout various stages of the pandemic engaged in higher levels of EHR-based after-hours work compared to those who used telemedicine less intensively. This suggests that telemedicine, as currently delivered, may be less efficient than in-person–based care and may increase the after-hours work burden of physicians.

## Introduction

The COVID-19 pandemic precipitated the rise of telemedicine—defined as the synchronous provision of health care services via telecommunications, either video or audio, to patients at remote sites—as a powerful disrupter of health care delivery [1-3]. Although not a new mode of work, the adoption and scaling of telemedicine prior to the pandemic was limited due to individual-, practice-, and system-level barriers that included technical and usability constraints, clinician practice patterns and preferences, security concerns, as well as payor and regulatory environments [4,5]. The significant disruptions to health care delivery caused by COVID-19 necessitated the rapid implementation of telemedicine in a variety of forms across practices and hospital systems in the United States and globally.

Prior to the pandemic, studies of the provision of clinical care through the medium of telemedicine identified potential benefits such as improved access to care in underserved regions or communities, better coordination of care, greater convenience, and lower costs [6,7]. Telemedicine may also have the potential to improve clinicians’ well-being and reduce burnout by improving associated risk factors such as on-call burden, communication, and job satisfaction [8-10]. At the same time, however, the introduction of novel technologies that impact the provision and experience of health care work can also be detrimental; in particular, there is concern about the impact of electronic health records (EHRs) on clinicians’ experience of work and its role in increasing both clinical and nonclinical administrative burden for physicians, including time spent on work-related tasks “outside” of clinical hours, often referred to as “work outside work” (WOW) or “pajama time” (PT) [11-14]. Shifting clinical and administrative work into personal time, particularly when physicians are at home, is a source of concern within the medical community, and it is unclear whether the proliferation of telemedicine as a form of health care work will exacerbate or ameliorate these conditions.

In this paper we focus on ambulatory physicians’ WOW during a time of rapid telework transition spurred by the COVID-19 pandemic. Our goal is to evaluate the impact of telemedicine practice on ambulatory physicians’ EHR-based WOW during the large-scale rollout of telemedicine in an urban academic hospital system during the COVID-19 pandemic.

## Methods

### Study Setting

New York University Langone Health (NYULH) is a large academic health care system in New York City, with over 8000 health care providers across 4 hospitals and over 500 ambulatory faculty group practices. The system is connected via a single EHR system, Epic, with over 7.5 million active patient accounts. Prior to the COVID-19 pandemic, NYULH offered limited telemedicine services only through pilot programs such as “virtual urgent care” (in emergency medicine), postoperative wound checks (in orthopedics), and some mental health services. Telemedicine for primary care and other routine health services was not available. During the pandemic, NYULH rapidly scaled its telemedicine offerings to include primary care, ambulatory specialty practice, and urgent care. NYULH “virtual health” was comprised of a single, enterprise-wide instance of synchronous, video-based telecommunications encounters between physicians and patients in remote locations accessed through a standardized EHR-based patient portal system and a third-party videoconferencing vendor. This platform provided a unified patient and provider experience between clinical practice sites and across specialties. At the height of the pandemic, this system saw an 8595% increase in monthly telemedicine visits between February (n=1699) and April (n=147,736), with over 2000 unique physicians engaging in video visits [15].

### Study Design

This is an EHR-based retrospective cohort study including all ambulatory care physicians continuously practicing (defined as at least 5 appointments scheduled per week in the reporting period) at any New York-based NYULH faculty group practice site between January 1, 2020, and August 31, 2020. Nonphysician practitioners (eg, advanced-practice providers) and residents were not included in the study cohort, as with few exceptions, they did not provide telemedicine-based care during this period.

### Ethical Considerations

This study was deemed part of a quality improvement and met the criteria for exemption from institutional review board’s review according to NYULH institutional policy. All data were collected as part of routine clinical care and administrative management.

### Study Measures

Definitions of key variables associated with study measures and analysis are provided in Table 1 and Table 2.

**Table 1:** Epic metric key terms and variables associated with study measures.

Epic metric	Description	Calculation
Reporting period	For a month, it starts on the Sunday on or immediately before the 1st and ends on the last Saturday of the month.	= (End date-start date)
Days with appointments	Percentage of days with at least one appointment within the reporting period.	For a reporting period: 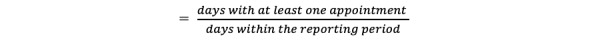
Appointments per day	Average minutes a provider spent in the system outside of scheduled hours.	For a reporting period: 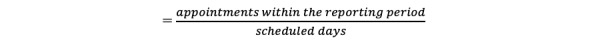
Time spent outside scheduled hours	Average minutes a provider spent in the system outside of scheduled hours.	For a reporting period: 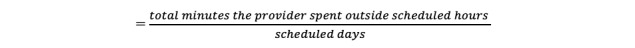
Time spent on unscheduled days	Average minutes a provider spent in the system on days with no scheduled patients.	For a reporting period: 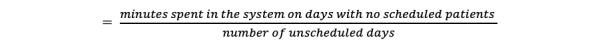

**Table 2:** Derived metric key terms and variables associated with study measures.

Derived metric	Calculation
Scheduled days	For a reporting period: 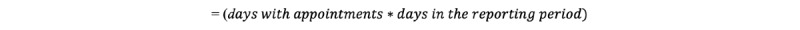
Unscheduled days	For a reporting period: 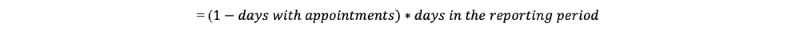
Time outside scheduled hours per month	For a reporting period: 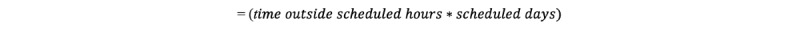
Time on unscheduled days per month	For a reporting period: 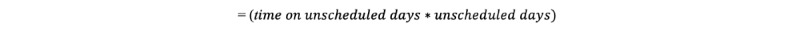
Clinical load	For a reporting period: 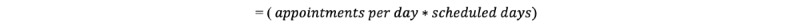
“Work outside work” measure	For a reporting period: 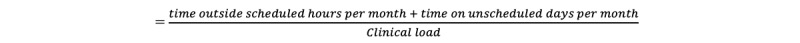

#### Pandemic Time Period

To evaluate whether the effects of telemedicine intensity were influenced by the evolving stages of the COVID-19 pandemic, we aggregated monthly physician data into the following 3 successive time periods: (1) the prepandemic period of January 1-February 29, 2020; (2) the acute pandemic period of March 1-May 31 (with March 15th representing the date when most NYULH ambulatory practices were closed for in-person visits); and (3) after the acute pandemic period of June 1-August 31, representing the gradual resumption of in-person care.

#### Telemedicine Intensity

To create a measure of the relative volume of clinical care physicians provided via telemedicine, we calculated the proportion of total visits per month that were telemedicine-based for each physician (number of video visits per month divided by the total number of all patient visits per month per provider) with values that could range from 0 to 1.

#### Clinical Load

Prior research has found clinical load to be an important predictor of WOW burden [11,14] and recommended normalizing WOW by load [11]. To account for the reduction and gradual resumption of in-person care during the pandemic, we created a measure of clinical load reflecting the total number of patient appointments for each physician each month. This was calculated by multiplying Epic-reported values of average number of appointments per clinical day (in-person or via telemedicine) by average number of clinical days per week, for each physician each month.

#### WOW

Derived from EHR user activity logs from Epic, WOW was calculated by adding time outside scheduled hours (ie, the average minutes per day spent in the system outside of scheduled hours on scheduled days, where scheduled hours are determined using Epic Cadence scheduling data plus two 30-minute “buffer” periods added before the start of first appointment and after the end of last appointment) and time on unscheduled days (ie, the average number of minutes per day spent in the system on days with no scheduled patients). WOW was normalized for physicians’ patient load by dividing WOW by clinical load to create a measure reflecting WOW per appointment.

An alternative measure of WOW uses the Epic EHR’s own variable-generated data—PT. PT represents the average number of minutes per day spent in charting activities on weekdays outside a standard (local) 7 AM to 5:30 PM workday and any time on weekends. PT does not include time spent personalizing EHR tools (eg, documentation templates or preferences lists) or time using reporting tools such as SlicerDicer and Reporting Workbench during unscheduled days. Although PT can be used as a marker of after-hours clinical work, recent studies have called into question its accuracy and usefulness for this purpose [15,16]. These concerns are likely exacerbated during the pandemic due to the significant disruptions in clinical care hours and work schedules for practices and physicians (eg, the closure of clinics, physician illness and exposure, and the variable outpatient work hours of physicians who were asked to provide emergency inpatient care), and therefore, this value was not included in this study.

### Statistical Analysis

We first computed telemedicine intensity, clinical load, WOW, and WOW per appointment for all physicians in the EHR that met our inclusion criteria. To evaluate whether WOW significantly varied across time periods, we ran one-way ANOVAs on both WOW and WOW per appointment. To evaluate the effect of telemedicine intensity and time period on after-hours work burden, as well as whether the relationship between telemedicine intensity and after-hours work varied across time periods, we conducted a hierarchical linear regression analysis in which the dependent variable was WOW per appointment. We first entered the main effects of telemedicine intensity and pandemic time period, followed by the interaction of telemedicine intensity and pandemic time period. To understand the nature of the interaction of telemedicine intensity and pandemic time period, we partitioned the data by time period and regressed WOW per appointment on telemedicine intensity in each time period. All analyses were conducted using SPSS (version 28; IBM Corp).

## Results

We analyzed data on 2129 physicians from January to August 2020. The majority of physicians were from internal medicine subspecialties (eg, cardiology, pulmonology, and geriatrics), followed by ambulatory surgery (including general surgery and surgical subspecialists) and general medicine practice (eg, internal medicine and family medicine; Table 3).

One-way ANOVAs evaluating whether the average WOW per day and WOW per appointment varied by pandemic time period were significant across physicians (average WOW per day: *F*_2_(2,12822)=33.09; *P*<.001; WOW per appointment: *F*_2_(2,12784)=42.68; *P*<.001). Average WOW per day declined during the acute pandemic relative to the prepandemic period and then reverted back to prepandemic levels after the acute pandemic. However, WOW per appointment increased during the acute pandemic period across all physicians, before subsequently declining (approaching but not reaching prepandemic levels) after the acute pandemic (Table 4).

**Figure 1:**
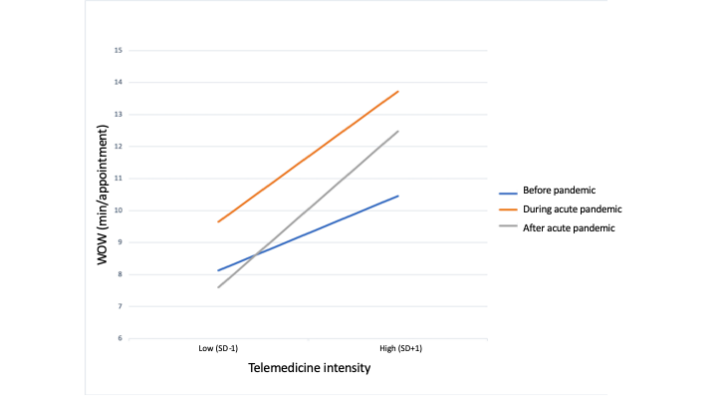
Work outside work (WOW) per appointment by telemedicine intensity and time period.

Across time periods (before the pandemic, during acute pandemic, and after acute pandemic) telemedicine intensity was positively associated with WOW per appointment (step 1 in Table 5), with physicians who spent a larger proportion of their time providing care via telemedicine devoting significantly more time to after-hours EHR work. Although the pandemic time period did not significantly affect WOW per appointment after controlling for telemedicine intensity, it significantly moderated the effect of telemedicine intensity on WOW per appointment (step 2 in Table 5). Regressions of WOW per appointment by telemedicine intensity for each time period showed that the positive relationship between telemedicine intensity and WOW per appointment was amplified over time, with the strongest positive relationship in the period after acute pandemic (Figure 1).

**Table 3:** Specialty of included study physicians (N=2129).

Clinical specialty	Values, n (%)
Internal medicine subspecialty	671 (31.5)
Surgery	377 (17.7)
General practice (eg, internal medicine and family doctors)	326 (15.3)
Pediatrics	175 (8.2)
Neurology	141 (6.6)
Obstetrician and gynecologist	134 (6.3)
Other	91 (4.3)
Psychiatry	72 (3.4)
Emergency medicine	68 (3.2)
Dermatology	36 (1.7)
Rehab	32 (1.5)
Pain medicine	6 (0.3)

**Table 4:** Work outside work (WOW) per day and per appointment, by time period.

WOW	Time period										
	Before pandemic				During acute pandemic				After acute pandemic		
	Median	Mean	95% CI	Median		Mean	95% CI	Median		Mean	95% CI
WOW per day	27.19	34.50	33.52-35.47	23.96		30.20	29.50-30.91	26.94		34.11	33.31-34.91
WOW per appointment	5.73	9.29	8.92-9.65	7.52		11.68	11.31-12.05	6.04		10.03	9.70-10.37

**Table 5:** Hierarchical regression of work outside work (WOW) per appointment.

Study variables	Normalized WOW						
	Step 1				Step 2		
	Unstandardized coefficient	Standard error	*P* value	Unstandardized coefficient		Standard error	*P* value
COVID-19 time period	–0.27	0.13	0.05	–0.52		0.15	<.001
Telemedicine intensity	6.67	0.32	<.001	1.37		1.41	
Telemedicine intensity×time period	N/A^a^	N/A	N/A	2.48		0.64	<.001

^a^N/A: not applicable.

## Discussion

### Principal Results

Our study found that telemedicine was less efficient than in-person–based care and increased physicians’ WOW burden. The overall EHR-based WOW declined for physicians in the context of the COVID-19 pandemic and the rapid transition to telemedicine; however, when controlling for changes in patient volume and clinical hours of care, physicians who devoted a higher proportion of their clinical time to telemedicine had higher levels of EHR-based WOW than those who used telemedicine less intensively. This relationship was present during all phases of the study (before the pandemic, during acute pandemic, and after acute pandemic) and was amplified over time, including in the after acute pandemic phase. These findings suggest that the observed decrease in the average WOW during the pandemic was the result of the overall decrease in clinical load for physicians rather than any benefits or efficiencies of telemedicine itself. Further, the amplification of the relationship between WOW per appointment and telemedicine intensity in the time period beyond the acute pandemic suggests that the WOW increasing effect of telemedicine was exacerbated over time, and therefore, the unique circumstances of the early COVID-19 pandemic alone are insufficient to explain the behavior patterns of physicians.

### Limitations

There are several limitations to this study that future research could address. First, limitations in our Epic-based data set preclude the ability to review and analyze physician EHR activity with sufficient granularity beyond certain time periods; for example, time periods more specific than a calendar month or physician activity log data at smaller than 15-minute increments. Specifically, Epic does not count WOW in its time outside of scheduled hours if that work occurs within the 30 minutes before or after patient scheduled hours (a “shoulder period”), which our analysis is unable to reliably differentiate as WOW time and therefore excludes, resulting in a systematic underestimation of the true WOW. Moreover, because shoulder time is added for each clinical day regardless of length, this underestimation bias is greater for physicians who spread their patient time over more scheduled days relative to those who see the same number of patients on fewer days [11]. Similarly, we are unable to target more specific times of pandemic disruption (eg, March 15, which is the exact date when most of our institution’s ambulatory clinics closed for in-person care). Second, we are limited in our ability to analyze activity at the level of physician or patient demographics; therefore, we are unable to comment on whether factors such as gender, age, or years in practice may have affected clinical load, telemedicine intensity, or WOW, and whether patient features such as patient complexity or acuity contributed to these outcomes. It is possible, for example, that telemedicine-based visits are overall less clinically intense compared to in-person visits due to differences in patient case mix, in which case our analysis would underestimate the time costs associated with telemedicine-based visits. Third, EHR-based data and work represent only part of the overall nonclinical burden of physicians. Additional time spent reviewing non-EHR based records, discussing care plans, working with interdisciplinary teams (eg, nurses and care managers), or advocating with insurers is not captured in this study; this work may have been increased during the pandemic due to disruptions in traditional office practices and workflows. Additionally, as our data are behavioral, we are unable to directly associate our measures with important factors such as physicians’ attitudes (eg, stress and burnout). Finally, our findings represent only the experience of physicians at a single health care system during the unusual period of the COVID-19 pandemic and the rapid transition to telemedicine, which may limit generalizability across provider type, practice environment, or geographic location.

### Interpretation of Findings in Clinical Context

To our knowledge, this is the first study to systematically evaluate the impact of the transition to telemedicine during the COVID-19 pandemic on physicians’ after-hours workload and one of a few studies that used EHR-based data to objectively evaluate after-hours work burden [15-17]. Although research documenting the experience of health systems undergoing the transition to telemedicine in response to the pandemic has increased [18-20], there is limited research exploring the effects of telemedicine on health care delivery areas such as clinical workflows, administrative load, or practice efficiencies, either during the pandemic or prior to it; the most robust of these works are almost a decade old and reflect a dated telemedicine environment that may no longer be relevant to the current context of health care delivery [21,22]. Similarly, literature exploring the impact of telemedicine on important aspects of physician work experience such as burnout and quality of life are limited, with the majority of work prior to the pandemic coming out of the field of telepsychiatry as an “early adopter” of the technology [23,24]. This study contributes to the literature on telemedicine in health care by exploring both the novel context of its expansion during the COVID-19 pandemic and its relationship to EHR-based work burden for clinicians.

A number of factors may be responsible for our findings that telemedicine increased the after-hours work burden of physicians. First, it is possible that organizational and technological inefficiencies in the early design, deployment, and scaling of telemedicine may have resulted in increased after-hours EHR work burden for physicians using telemedicine more intensively. These include early and ongoing technological issues relating to the computer hardware, software functionality and integrations, and user experience of the “virtual health” platform deployed by our system. These issues have been highlighted elsewhere in EHR and digital health technology implementation research, particularly regarding usability and user experience barriers [25-27] exacerbated by the scale and abruptness of the transition to telemedicine due to the pandemic [28]. However, technological inefficiencies should be at least partially ameliorated over time as physicians learn to navigate and optimize their setup and systems (the “learning curve”), an assumption that is not supported by our after acute pandemic period findings of a continued “amplified” relationship between telemedicine intensity and after-hours EHR work. Similarly, telemedicine training for physicians during this period of rapid expansion was often ad hoc and likely suboptimal for the development of effective telemedicine competencies (eg, efficient platform navigation, technical troubleshooting, “virtual health” EHR documentation), and thereby, potentially worsening WOW; however, this would be expected to improve with time as physicians adapted their workflows and learned new skills, rather than, as our results found, establishing a pattern of increasing work burden in the later periods of telemedicine of use, even as access to quality telemedicine trainings and best practice knowledge sharing improved among institutions. This suggests that “virtual health” training as it existed during the early phases of the pandemic was not sufficient to improve after-hours work burden for physicians. Further exploration of the relationship between telemedicine training and “virtual health” practice patterns (including EHR-based activities) is warranted as training becomes more regularly integrated into medical education and professional development.

The second factor that might have impacted our findings is that it is likely that significant disruptions to the work norms of clinical practices during the pandemic affected after-hours work patterns. In clinics, individual- and practice-level adjustments to the demands of care provision during the pandemic likely resulted in a number of unique work structures and arrangements that could have likely affected physicians’ work schedules, including time spent doing after-hours work. In particular, the shift to a telemedicine-based platform—particularly one with limited multiparty functionality—may have inhibited effective team-based care between physicians and clinical support staff (eg, medical assistants) and shifted both clinical and administrative tasks that had prior been completed by other staff members onto physicians. This “doctor does it all” phenomenon has been recently described as an unintended effect of the rapid transition to telemedicine during the pandemic [29]; within our own system, much of the current WOW involves responding to patient messages, phone calls, refill requests, and completing various EHR documentation requirements often left for the end of the day after direct patient care responsibilities are ended. NYULH is actively engaged in reducing this burden on providers by redistributing relevant work to support staff, as well as using novel technologies including machine learning to facilitate message triage and management, for example by suppressing messages that are not actionable by providers. More work is needed to fully understand the impact of the new virtual-first models of care delivery on interdisciplinary teams and team-based practice.

### Learning from Other Fields and Implications for Health Care Practice

Overall, our results suggest that telemedicine is not panacea for the work challenges facing clinicians. In fact, our evidence during the acute pandemic and after the acute pandemic suggests that rather than reducing administrative burden, telemedicine intensity may increase it, shifting the work temporally and spatially to after-hours work and home. This suggests that a more thorough understanding of the implications of telemedicine in clinical practice is necessary prior to its indiscriminate expansion to ensure policies and practices that increase efficiency and work-life quality and counteract inefficiencies, waste, and work-related stress and burnout are implemented. Given the limited data available on the impact of telemedicine on important aspects of physicians’ experience of work, it may be instructive to look to fields outside of medicine, where the study of “telework” (defined as a work arrangement that allows employees to perform work at approved alternative or remote worksites) [29] is more robust. Research in the industries of engineering, consulting, and software development has demonstrated varying effects of remote work on key elements of employees’ work experience. Positive effects of telework in these fields include increased job satisfaction, performance, and work-life balance, as well as reduced employee turnover, real estate costs, commute time, and environmental impact [30-32]. Conversely, negative effects include reduced career development and feelings of reduced energy, confidence, and engagement due to the loss of high-quality interaction with colleagues and clients [33,34]. Significantly, telework has been associated with workers’ inability to disconnect from their work and increased stress-inducing work intensification [35,36]. This relationship may apply to telemedicine and help explain the findings in this study. Although more investigation is needed to understand the full scope and implications of medical telework beyond the direct care provided by telemedicine (including tasks such as remote teaching, non-EHR–based clinical work, administrative work, and research), general learning from these fields may help identify and guide key areas of future telemedicine and telework research.

### Conclusions

In this study, we evaluated the impact of the transition to telemedicine during the COVID-19 pandemic on physicians’ EHR-based after-hours workload; we found that when controlling for the clinical load of patient visits, physicians who devoted a higher proportion of their clinical time to telemedicine engaged in higher levels of EHR-based after-hours work compared to those who used telemedicine less intensively; this relationship persisted and was amplified over time, even after the acute pandemic period. This suggests that telemedicine, as currently delivered, may be less efficient than in-person–based care and may contribute to after-hours work burden of physicians. Further study is needed on the detailed impacts of telemedicine on physician work practices, particularly in contexts beyond the COVID-19 pandemic and relating to administrative burden, after-hours clinical responsibilities (particularly the EHR-related in-basket and patient portal messaging responsibilities), and experience of work. Learning from other industries where telework is more established can help identify areas of need and opportunity in future telemedicine care delivery.

## Abbreviations

EHR: electronic health record
NYULH: New York University Langone Health
PT: pajama time
WOW: work outside work

## Data Availability

The data underlying this article will be shared upon reasonable request to the corresponding author.
